# Mechanobiological Principles Influence the Immune Response in Regeneration: Implications for Bone Healing

**DOI:** 10.3389/fbioe.2021.614508

**Published:** 2021-02-12

**Authors:** Raphael S. Knecht, Christian H. Bucher, Sophie Van Linthout, Carsten Tschöpe, Katharina Schmidt-Bleek, Georg N. Duda

**Affiliations:** ^1^Julius Wolff Institute and Center for Musculoskeletal Surgery, Charité—Universitätsmedizin Berlin, Berlin, Germany; ^2^Berlin Institute of Health Center for Regenerative Therapies, Charité—Universitätsmedizin Berlin, Berlin, Germany; ^3^German Center for Cardiovascular Research (DZHK), Partner Site Berlin, Berlin, Germany; ^4^Department of Cardiology, Charite'—Universitätsmedizin Berlin, Berlin, Germany

**Keywords:** regeneration, inflammation, mechanobiology, mechano-transduction, immune-mechanics, YAP/TAZ, TRPV4, PIEZO1

## Abstract

A misdirected or imbalanced local immune composition is often one of the reasons for unsuccessful regeneration resulting in scarring or fibrosis. Successful healing requires a balanced initiation and a timely down-regulation of the inflammation for the re-establishment of a biologically and mechanically homeostasis. While biomaterial-based approaches to control local immune responses are emerging as potential new treatment options, the extent to which biophysical material properties themselves play a role in modulating a local immune niche response has so far been considered only occasionally. The communication loop between extracellular matrix, non-hematopoietic cells, and immune cells seems to be specifically sensitive to mechanical cues and appears to play a role in the initiation and promotion of a local inflammatory setting. In this review, we focus on the crosstalk between ECM and its mechanical triggers and how they impact immune cells and non-hematopoietic cells and their crosstalk during tissue regeneration. We realized that especially mechanosensitive receptors such as TRPV4 and PIEZO1 and the mechanosensitive transcription factor YAP/TAZ are essential to regeneration in various organ settings. This indicates novel opportunities for therapeutic approaches to improve tissue regeneration, based on the immune-mechanical principles found in bone but also lung, heart, and skin.

## Introduction

The immune response is essential for successful healing after an injury. Even under sterile conditions, the immune system reacts to injury with a pro-inflammatory reaction as a first step (Midwood and Piccinini, 2010; Schmidt-Bleek et al., 2012, 2015). However, a misguided immune cell response leading to a too pronounced pro-inflammatory response or a prolonged anti-inflammatory response ends in an unsuccessful or delayed healing outcome (Wynn, 2004; Braga et al., 2015; Schmidt-Bleek et al., 2015; Dellacherie et al., 2019). Until now, research has mainly focused on how different cytokines and other small soluble molecules influence the local immune response and subsequently the healing outcome. According to Wolff's law, tissue adaptations in bone in particular are due to physical or mechanical forces during its dynamic homeostatic remodeling (Wolff, 1986; Rho et al., 1998). This force-sensitivity is also remarkably relevant for healing and regeneration such as the healing of bone fractures (Lienau et al., 2005; Schell et al., 2005; Epari et al., 2007; Ghiasi et al., 2017). For example osteocytes show extraordinary mechano-sensitivity and are engaged in regulating other cells in their activity (e.g., osteoblasts and osteoclasts) according to their mechanical sensation they perceive (Bonewald and Johnson, 2008; Prideaux et al., 2016; Qin et al., 2020; Sato et al., 2020). It is becoming increasingly apparent how relevant the crosstalk between immune cells and bone cells is in both homeostasis and regeneration (Tsukasaki and Takayanagi, 2019). However, how the cell niche and its surrounding, notably its mechanical properties, geometry and constitution, are involved in the crosstalk between non-hematopoietic and immune cells has long been overlooked or neglected.

Apparently, the control of cellular responses and cell behavior via mechanical cues is essential for the homeostasis and regeneration of tissue. Therefore, mechanical signals hold great potential for new therapeutic approaches, and they have inherent properties that circumvent some challenging and often application-limiting problems associated with small molecules or proteins. For example, mechanical cues only act locally, and thus, there is no diffusion of the therapeutic stimuli. As a result, mechanical stimuli can, if correctly designed and as needed, remain within the “therapeutic window” for days, weeks, months, or even years and without the need for constant re-application of the therapeutic agent. Moreover, when biomaterials are implanted *in vivo*, they are not mechanically isolated but come into contact with the surrounding tissue and cells. Therefore, forces, pressure, deformations and fluid flow should be considered as potential features for any therapeutical use of mechano-biological cues in addition to the inherent mechanical properties of the biomaterial such as stiffness, (visco-)elasticity, pore size, patterns, shape, and geometrical organization. Interestingly, matching for example the stiffness of the biomaterial to the surrounding tissue could already minimize the foreign body response (Carnicer-Lombarte et al., 2019). Deciphering such mechanical interfaces between biomaterials, surrounding tissue and immune cells will enable new modulation and intervention strategies in regenerative medicine.

So far, the exact mechanism and receptors involved in the perception of the mechanical niche around the cells and how this alters cell behavior have not yet been fully elucidated. This review addresses the question how relevant the mechanical niche is for immune cells in tissue regeneration. We aim to review what is known for different tissue regeneration processes as they are known in bone, lung, heart and skin. With such an approach we intend to decipher the immunomodulatory mechanical features that are either directly perceived by the immune cells themselves or indirectly by non-hematopoietic cells. In the latter case, non-hematopoietic cells act as carriers of mechanical properties to immune cells. Understanding critical mechanical properties that either induce pathological processes in the body or initiate a successful healing response will help to exploit the full potential of mechanical regulation of cell behavior *in vivo* for future therapeutic approaches.

**Figure 1:**
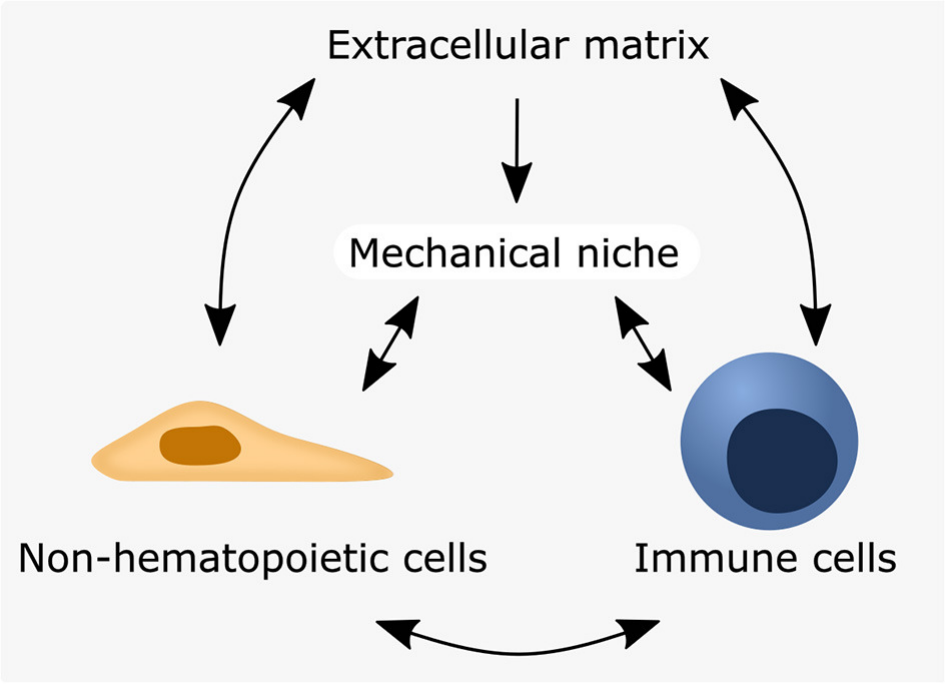
The mechanical niche defined by the extracellular matrix influences the crosstalk between non-hematopoietic cells and immune cells.

This review highlights the close crosstalk between the immune system and non-hematopoietic cells during pathological and regenerative processes in bone tissue, with a focus on mechanical constraints. We suggest that the mechanical niche influences ECM composition, non-hematopoietic and immune cells and thereby changes their crosstalk (Figure 1). Bone is of particular interest because it is able to regenerate without scarring, which represents the “ideal” healing capacity. At present, studies focusing on mechanical properties that influence crosstalk between immune cells, non-hematopoietic cells and the ECM in the development of pathologies or regeneration in bone are relatively rare. Therefore, we have looked at other tissues such as the lung and the heart, where more studies have already been conducted to decipher the crosstalk between immune cells, non-hematopoietic cells and the ECM from a mechanical point of view. We also looked at skin tissue, which is often used as a model to study wound healing. Finally, we will look back at the bone to identify common principles of interaction between the immune system and the mechanical niche and will point to possible new research opportunities.

## Bone Healing Is Modulated Via Immune-Mechanics

### Bone Cells Are Influencing Immune Cell Development

The bone marrow harbors the hematopoietic stem cell (HSC) niche, which gives rise to the myeloid and lymphoid compartment of the immune system (Okamoto et al., 2017). Besides this spatial proximity between the HSC niche and bone, they are also developmentally and functionally connected. Osteoclasts are derived from monocytes in the myeloid compartment of the immune system, while mesenchymal stem cells (MSCs) within the bone marrow give rise to osteoblasts, and also contribute to the HSC niche (Loi et al., 2016). Even after their development from common progenitors, bone and immune cells stay in close contact with each other and more and more mechanisms have now been discovered about how bone cells influence immune regulation and homeostasis (Takayanagi, 2007).

Osteoblasts, osteocytes and osteoclasts have all been described to influence immune cells in physiological and pathological conditions (Tsukasaki and Takayanagi, 2019). HSCs must remain in the bone marrow niche to maintain their stem cell status and to allow the continuous formation of new blood and immune cells (Suárez-Álvarez et al., 2012; Morrison and Scadden, 2014). On the other hand, however, the recruitment of immature and maturing hematopoietic cells into the periphery has been suggested to contribute to host defense and tissue repair (Cottler-Fox et al., 2003; Kollet et al., 2003).

Osteoblasts progenitors, more specifically CXC-chemokine ligand 12 (CXCL12) abundant reticular (CAR) cells, were shown to contribute to the HSC niche by expressing CXCL12, a chemokine with important functions in the homing and retention of hematopoietic stem/progenitor cells in the bone marrow (Lévesque et al., 2003; Omatsu et al., 2010; Greenbaum et al., 2013). Moreover, CXCL12 from osteoblast progenitors plays an important role for normal B- and T cell development in the bone marrow (Ara et al., 2003; Greenbaum et al., 2013; Tsukasaki and Takayanagi, 2019). The dysregulation of osteoblasts via the overexpression of jagged 1 has been associated with acute myeloid leukemia in mice (Kode et al., 2014). Moreover, sepsis-induced osteoblast ablation has been shown to lower interleukin (IL)-7 levels in the hematopoietic stem cell niche, resulting in lymphopenia and immunodeficiency (Terashima et al., 2016).

Osteocytes were shown to regulate hematopoiesis and myeloid expansion via the secretion of the proliferative factor granulocyte colony-stimulating factor (G-SCF) (Fulzele et al., 2013). Moreover, the ablation of osteocytes in mice was associated with defects in bone marrow, thymus and spleen and subsequently showed sever lymphopenia (Sato et al., 2013). In bone, osteocytes are the main producer of sclerostin, a potent inhibitor of osteoblastic bone formation (Li et al., 2008). Sclerostin-knockout mice however, showed also a depletion of the B cell lineage within the bone marrow and significant reduction of CXCL12 (Cain et al., 2012).

Osteoclasts have been shown to secrete matrix metallopeptidase 9 (MMP9) and cathepsin K, both playing a role in the degradation and inactivation of CXCL12 (Kollet et al., 2006). In addition, the administration of osteoclast stimulator receptor activator of nuclear factor (NF)-κB (RANK) ligand (RANKL) led to a decrease in CXCL12 and promoted the mobilization of hematopoietic progenitor cells, thereby impairing hematopoiesis (Kollet et al., 2006). Moreover, high calcium concentration produced during bone resorption via osteoclasts has been described to be essential for HSCs niche maintenance (Adams et al., 2006).

Thus, bone progenitor cells and mature bone cells are important for the regulation and maintenance of the HSC niche. Consequently, dysfunctional bone (progenitor) cells in the bone marrow have been found to be associated with immune system pathologies.

### Immune Cells Are Regulating the Activity of Bone Cells

Conversely, both lymphoid and myeloid immune cells influence the behavior of bone cells in many bone pathologies by altering the balance of bone formation and resorption (Tsukasaki and Takayanagi, 2019).

Bone resorption is tightly regulated via the RANKL-RANK system (Boyce and Xing, 2008). Signaling via RANKL-RANK together with macrophage colony-stimulating factor (M-CSF) is needed for the maturation and activation of osteoclast precursors and thus promotes bone resorption (Kong et al., 1999; Okamoto et al., 2017). Osteoprotegerin (OPG) is produced by B cells, dendritic cells, MSCs and osteoblasts and can act as decoy receptor for RANKL, blocking RANK mediated signaling in osteoclast precursors and reduce their maturation and activation, leading indirectly to more bone formation (Khosla, 2001). Moreover, T cells also express RANKL and may thereby promote osteoclast maturation and increase bone loss (Theill et al., 2002; Takayanagi, 2007; Weitzmann, 2013). However, the effect of T cells expressing RANKL is often overridden by the secretion of interferon (IFN)-γ by T helper (TH) 1 cells (Takayanagi et al., 2000). IFN-y interferes with the RANKL-RANK signaling pathway and thus reduces osteoclastogenesis (Takayanagi et al., 2000). While IL-6 secreted by T_H_2 was found to increase RANKL expression on MSCs and osteoblasts, IL-4 secreted by T_H_2 has been shown to interfere with the RANKL-RANK signaling pathway and reduce osteoclastogenesis (Takayanagi, 2007; Claes et al., 2012). T_H_17 cells have been shown to promote osteoclastogenesis in rheumatoid arthritis mainly by promoting RANKL expression in synovial fibroblasts via IL-17 secretion (Sato et al., 2006; Danks et al., 2016).

In addition to the lymphoid cells of the immune system, the myeloid compartment has also been described to regulate bone homeostasis (Loi et al., 2016). Pro-inflammatory cytokines including tumor necrosis factor (TNF), IL-1 and IL-6 secreted by macrophages promoted osteoclastogenesis and increased bone resorption (Boyle et al., 2003; Takayanagi, 2007). However, monocytes and macrophages also support osteoblast differentiation and proliferation through the secretion of bone morphogenetic protein (BMP)-2, BMP-4 and transforming growth factor (TGF)-b1 (Fromigué et al., 1998; Champagne et al., 2002; Blom et al., 2004). Furthermore, macrophages promote mineralization and osteogenic differentiation of MSCs via b-tricalcium phosphate and oncostatin m (Chen et al., 2014; Guihard et al., 2015). In addition, the polarization of macrophages toward an M2 phenotype via the administration of IL-4 and IL-13 has been shown to promote the mineralization of the soft callus after bone fracture (Schlundt et al., 2018).

These findings suggest that the number and activity of osteoclasts and osteoblasts can be regulated by lymphoid and myeloid immune cells and therefore shift the balance between bone resorption and bone formation.

### Immune Cells Are Crucial Players for Successful Bone Fracture Healing

The close interaction of bone and immune cells already suggests that the immune system also plays a decisive role in bone fracture healing. Indeed, timing of the initial pro-inflammatory and subsequent anti-inflammatory phase during bone healing is a necessity for successful healing (Mountziaris and Mikos, 2008; Schmidt-Bleek et al., 2012, 2014, 2015; Spiller et al., 2015; Maruyama et al., 2020).

The healing cascade after bone fracture can be divided into consecutive but partly overlapping phases (Kolar et al., 2010; Schmidt-Bleek et al., 2015). During the fracture event, blood vessels are disrupted, resulting in hematoma formation. This early fibrin network marks the start of the inflammatory phase and creates a first provisional matrix, into which mainly pro-inflammatory polymorphonuclear leukocyte (PMNs) migrate in and secrete pro-inflammatory cytokines, such as C-C motif chemokine 2 (CCL2) and IL-6 (Yang et al., 2007; Xing et al., 2010; Bastian et al., 2011, 2016). The immigrated PMNs secret CCL-2 and IL-6 recruit, which attracts macrophages and monocytes from the surrounding tissue and vessels into the hematoma (Hurst et al., 2001; Xing et al., 2010; Claes et al., 2012). The recruited macrophages start to phagocytose necrotic cells and debris and secrete further pro-inflammatory cytokines such as TNF, IL-1β, IL-6, and CCL2, which recruits fibroblasts, MSCs and osteoprogenitor cells (Kon et al., 2001; Bielby et al., 2007; Wu et al., 2013).

In addition, lymphoid cells have also been found to immigrate during the pro-inflammatory phase into the fracture hematoma (Glynne Andrew et al., 1994; Könnecke et al., 2014). Pro-inflammatory factors such as IL-1, IL-6, and TNF have been found to increase RANKL expression on activated T cells, resulting increased osteoclastogenesis (Clowes et al., 2005; Hardy and Cooper, 2009). Even though, activated T cells also secrete interferon (INF)-γ, that interferes with RANKL signaling through downregulating tumor-necrosis factor-receptor-associated factor 6 (TRAF6), it has been suggested, that RANKL mediated promotion of osteoclastogenesis is dominant over the IFN-γ mediated downregulation of osteoclastogenesis (Takayanagi, 2007; Greenblatt and Shim, 2013). Moreover, IFN-γ secreted by cytotoxic T cells also led to a higher pro-inflammatory M1 macrophage polarization (Schmidt-Bleek et al., 2012). Thus, IFN-γ seems to have more pro-inflammatory and osteoclast promoting functions.

This first pro-inflammatory phase, characterized by the formation of the hematoma and recruitment of pro-inflammatory cells, has been found to be critical for a successful healing outcome (Gerstenfeld et al., 2003; Yang et al., 2007; Kolar et al., 2010; Glass et al., 2011). However, within the inflammatory phase, an anti-inflammatory response is needed to resolve inflammation and allow for the transition to the subsequent soft callus and hard callus phase (Serhan and Savill, 2005; Schmidt-Bleek et al., 2015). IL-4, IL-10, and TGF-β secreted by regulatory T cells (Tregs) interfere with RANKL signaling and reduce osteoclastogenesis and thus the presence of Tregs were associated with a shift from pro- to an anti-inflammatory phase (Arron and Choi, 2000; Takayanagi et al., 2000). In the soft callus phase, connective tissue is formed first, and later replaced by the formation of cartilage via the differentiation of MSCs into chondrocytes (Gerstenfeld et al., 2003; Claes et al., 2012). This soft callus gives initial mechanical stability and serves as a scaffold for new bone formation via endochondral ossification during the hard callus phase, where MSCs and osteoprogenitors are recruited, differentiate into osteoblasts, and start to mineralize the ECM, which can be remodeled to regain form and function of the bone tissue (Bielby et al., 2007; Loi et al., 2016).

#### Immuno-Modulatory Interventions to Promote Bone Healing

Both the pro- and anti-inflammatory phases are necessary for successful healing and excessive downregulation of either phase can inhibit the healing cascade (Schmidt-Bleek et al., 2015; Rapp et al., 2016). Thus, factors influencing either the pro-inflammatory or anti-inflammatory phase have been shown to largely affect the fracture healing outcome. Most often, the aim of the intervention lays on re-establishing the balance between pro- and anti-inflammatory response, with a focus on preventing a prolonged pro-inflammatory phase.

In mice for example, the transplantation of MSCs to mediate bone repair, was found to be considerably improved with additional downregulation of TNF-α and IFN-γ via the administration of aspirin or systemic infusion of Tregs (Liu et al., 2011). The authors propose that IFN-γ, secreted by the recipients T-helper cells, downregulate runt-related transcription factor 2 (Runx-2) in the transplanted MSCs and thus inhibits the osteogenic differentiation (Liu et al., 2011).

The administration of a prostacyclin (PGI_2_) analog has been shown to improve fracture healing by downregulating pro-inflammatory cytokine release from CD8+ and CD4+ T cells while promoting the anti-inflammatory response via an increased numbers of M2 macrophages in the fracture gap (Wendler et al., 2019). Similarly, Tregs are usually associated with anti-inflammatory functions and have been shown to inhibit osteoclastogenesis by cell-cell contact and the expression of transforming growth factor-beta (TGF-β), IL-4, and IL-10 (Zaiss et al., 2007). Consequently, the adoptive transfer of Tregs improved fracture healing, when they overcome a certain CD8+ effector T (T_eff_) cell/Treg ratio (Schlundt et al., 2019). Additionally, an elevated concentration of terminally differentiated CD8+ effector memory T cells (CD8+CD11a++CD57+CD28-T cells), a subpopulation known for high pro-inflammatory properties, at the fracture gap significantly delayed the fracture healing cascade in patients. While the depletion of CD8+ T cells by specific antibody therapy was shown to enhance the endogenous fracture regeneration in a murine model, an adoptive transfer of CD8+ T cells however impaired the fracture repair (Reinke et al., 2013). Higher concentration of effector memory T cells can be found in a more experienced immune system, which usually has a higher propensity for pro-inflammatory effects (Bucher et al., 2019).

Therefore, gaining control over the immune system may lead to promising therapeutic approaches in bone pathologies and regeneration.

### Mechanical Properties Are Regulating the Immune Response Following Fracture

Until now, therapeutic approaches have mostly focused on biological factors that influence the immune response. Thus, it has mostly been overlooked so far, how different biophysical and mechanical properties during the healing cascade might directly or indirectly influence the immune response and subsequently suppress or promote bone healing.

At the tissue level, the stability of the fracture gap seems to be closely related to the healing outcome. A stable fracture fixation (minimizing interfragmentary movement) most likely leads to direct bone formation, a moderately fixed fracture may heal by endochondral ossification and an unstable fixed fracture gap almost suppresses the bone healing cascade (Epari et al., 2006, 2007; Claes et al., 2012). How are these mechanically different situations related to the healing process? At the cellular level, cyclic tensile strain applied to osteoblasts was found to induce higher OPG and lower RANKL expression (Tang et al., 2006). Conversely, dynamic compression of osteoblasts *in vitro* showed higher expression of pro-inflammatory IL-6, IL-8, RANKL, and lower expression of OPG, possibly delaying a bone healing response by increased osteoclast activity (Sanchez et al., 2009, 2012). Moreover, compression of osteoblasts stimulated expression of the IL-17 gene. IL-17 has been shown to increase the expression of IL-1, IL-6, IL-8, IL-11 in osteoblast cells in an autocrine manner (Zhang et al., 2010). A function of IL-17 is to stimulate the expression of RANKL on osteoblasts, thereby increasing osteoclastogenesis and osteoclast activity, which may eventually lead to bone erosion (Miossec and Kolls, 2012). Furthermore, systemically elevated levels of IL-8 have been found after bone fracture, and it has been shown that mechanically stimulated osteoblasts release IL-8, which may be the source of the systemically elevated IL-8 levels (Perl et al., 2003; Reinke et al., 2013). IL-8 appears to be involved in several regenerative processes, such as the regulation of revascularization of newly formed tissue after a fracture (Koch et al., 1992; Hou et al., 2014). A lack of sufficient revascularization has been associated with poorer healing outcome (Schmidt-Bleek et al., 2015). In addition, IL-8 is also a chemokine, that attracts polymorphonuclear leukocytes (PMNs) to the site of inflammation (Kolar et al., 2011; Vasconcelos et al., 2016). The attraction of PMNs to the fracture, does not *per se* negatively affect the healing outcome, since an initial pro-inflammatory response is required for a good healing outcome (Schmidt-Bleek et al., 2015). Moreover, IL-8 has been shown to be important for stem cell recruitment and increased chondrogenic gene expression and bone morphogenetic protein (BMP) sensitivity, thereby positively influencing osteochondral bone healing (Lin et al., 2019). It appears, that at least the osteoblasts are actively involved in the perception of cyclic stretch and respond to it by secreting pro-inflammatory cytokines. Moreover, osteoblast proliferation has been found to occur mostly during the pro-inflammatory phase (Gerstenfeld et al., 2003; Watanabe et al., 2009)

Beyond the above-mentioned aspects, little is known about the regulation of the immune-mechanical interplay in bone healing. However, in other organ systems a more profound knowledge on mechano-immunological coupling has been gathered, which we would like to compare with the bone-related knowledge in order to gain a more comprehensive understanding on how immune-mechanics influences tissue regeneration. Although lung, heart, and skin tissue seem at the first very different, the universal feature that we think allows for comparison and extrapolation of information to bone tissue is their remarkable mechanosensitivity, constant exposure to high forces and stress, and of course all tissues are infiltrated by the immune system in health and disease.

## Lung Inflammation Following Injury Is Modulated Via Immune-Mechanics

**Figure 2:**
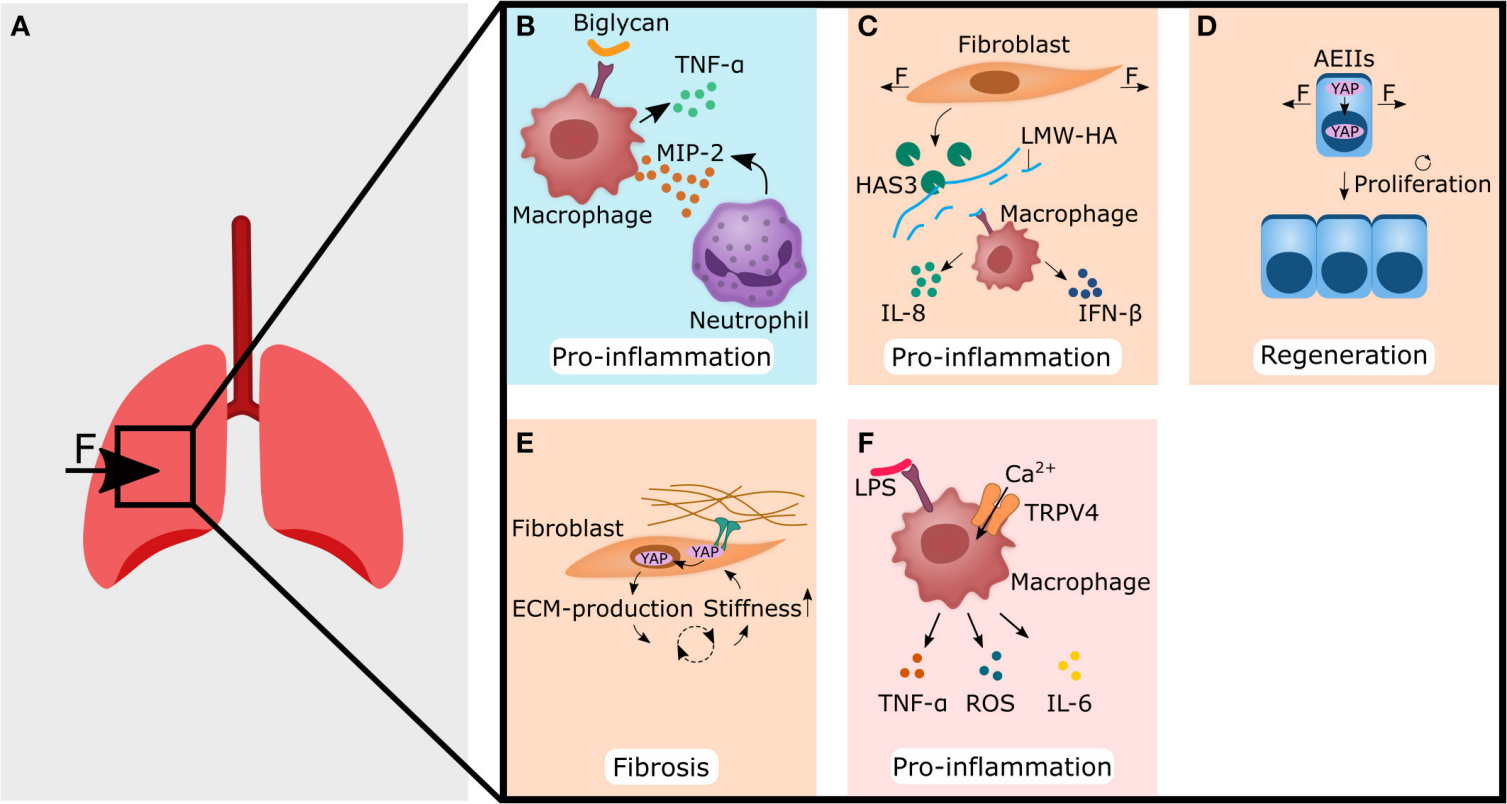
Forces acting on lung tissue changes the extracellular matrix (blue panel), or are perceived by non-hematopoietic (light orange panels) and immune cells (pink panel), thereby setting up fibrosis and pro-inflammatory responses. **(A)** Forces that are caused for example by mechanical ventilation act on lung tissue. This may lead to the cellular responses shown in **(B–F)**. **(B)** Biglycan binds to TLR4 receptor on macrophages, thereby leading to the secretion of TNF-α and MIP-2. The latter acts as a chemoattractant for neutrophils. **(C)** Stretching of fibroblasts increases the amount of hyaluronan synthase 3 (HAS3), which produces low molecular weight hyaluronic acid (LMW-HA). Macrophages then bind LMW-HA via TLR4 and react with the secretion of IL-8 and IFN-β, both of which are associated with pro-inflammatory functions. **(D)** Cyclic stretch of alveolar epithelial type II cells (AEIIs) leads to nuclear localization of YAP/TAZ, and subsequently increases the proliferation rate. **(E)** Increased stiffness of the extracellular matrix (ECM) leads to nuclear localization of YAP in fibroblasts, which subsequently show enhanced production of ECM components. This might increase the stiffness of the ECM again and start a vicious cycle, that may end in fibrosis. **(F)** Calcium influx in macrophages via TRPV4 and crosstalk with LPS mediated signaling leads to increased secretion of pro-inflammatory cytokines such as TNF-α, ROS, and IL-6. The YAP symbol in the illustrations is representative for YAP and TAZ. CXCL, C-X-C motif ligand; IL, interleukin; IFN, interferon; LPS, lipopolysaccharides; MIP-2, macrophage inflammatory protein-2; TAZ, WW domain-containing transcription regulator 1; TNF-α, tumor necrosis factor alpha; TRPV4, transient receptor potential vanilloid 4; YAP, yes-associated protein 1.

Positive pressure mechanical ventilation is a life-saving therapy for patients with acute lung injury (ALI), or its more severe form, acute respiratory distress syndrome (ARDS) (Kuipers et al., 2011). However, it can also lead to ventilator-induced lung injury (VILI), which exacerbates pre-existing lung damage and inflammation (Tremblay and Slutsky, 2006; Kuipers et al., 2012) (Figure 2A). To reduce the release of inflammatory mediators, it has been suggested that mechanical ventilation with lower tidal volumes should be preferred over high tidal volumes, even though this may cause acidosis and reduced arterial oxygenation (Blanch et al., 1994; Brower et al., 2000). A deeper understanding of how mechanical overdistension of the lung causes inflammatory processes is therefore highly desirable to minimize the side effects of mechanical ventilation in patients with already severe conditions. Moreover, the mechanisms we may learn from understanding how mechanical ventilation induces pro-inflammatory reactions, is an excellent tool to decipher immune-mechanical principles.

### ECM-Components as Mechano-Transducer in Inflammation

One possible way how mechanical stimuli can alter the immune response is by changing the ECM-composition. It has been shown, that excessive mechanical ventilation increased the amount of proteoglycan and biglycan in the lung (Al-Jamal and Ludwig, 2001). Biglycan was found to bind to the Toll-like receptor (TLR) 4 on macrophages and to increase the expression of pro-inflammatory TNF-α and macrophage inflammatory protein (MIP)-2 (also known as CXCL2, a chemokine/cytokine sharing the same receptor with human IL-8), thereby triggering or promoting an already ongoing inflammatory response (Schaefer et al., 2005) (Figure 2B).

Another ECM component often associated with trauma is hyaluronic acid (HA). HA exists as a more immunological inert high molecular weight form (HMW-LA), and as pro-inflammatory low molecular weight hyaluronic acid (LMW-HA) form. Mechanical ventilation of rat lungs with high tidal volumes but not low tidal volumes led to an accumulation of LMW-HA in lungs (Mascarenhas et al., 2004). On a cellular level, stretching of pulmonary fibroblasts or alveolar epithelial type II cells (AEIIs) induced *in vitro* the expression of hyaluronan synthase 3 (HAS3); the enzyme that produces LMW-HA (Itano et al., 1999; Mascarenhas et al., 2004; Heise et al., 2011). LMW-HA might then bind to TLR2 or TLR4 on macrophages and increase the expression of IL-8 and IFN-β and finally induces a pro-inflammatory response after mechanical ventilation with high tidal volumes (Jiang et al., 2005; Black et al., 2013) (Figure 2C). Moreover, LMW-HA has been shown to be sufficient for inducing epithelial to mesenchymal transition (EMT) of AEIIs, often associated with aberrant wound healing and fibrosis (Heise et al., 2011). Interestingly, stretched fibronectin conformation has been found to bind to TLR4 on lung cancer cells and increased IL-8 expression and release (Cho et al., 2020).

This suggests that not only the presence of fibronectin, but also the mechanical environment and consequently the conformation of fibronectin may have important implications. A stretched fibronectin conformation could be an indicator of mechanically stressed tissue and could be involved in triggering inflammation by increasing the levels of IL-8.

### Non-hematopoietic Cells as Mechano-Transducer in Inflammation and Regeneration

The yes-associated protein 1 (YAP)/ WW domain-containing transcription regulator 1 (WWTR1, also known and more commonly referred to as TAZ) pathway integrates mechanical stimuli, soluble signals, and metabolic information to regulate cell proliferation, cell plasticity and stemness (Totaro et al., 2018; Pocaterra et al., 2020). Although not exclusively regulated by mechanical stimuli, the YAP/TAZ pathway has been shown to be highly mechanosensitive (Dupont et al., 2011). YAP/TAZ can translocate into the cell nucleus by cell-cell contact signaling and/or ECM-integrin-focal adhesion kinase (FAK) signaling (Dupont et al., 2011; Pocaterra et al., 2020) or forces can directly trigger the entry of YAP into the cell nucleus by regulating transport through nuclear pores (Elosegui-Artola et al., 2017). Additionally, cyclic stretch has been shown to increase YAP nuclear localization in AEIIs and subsequently their proliferation rate (Codelia et al., 2014) (Figure 2D). This could have a significant impact on lung regeneration, as ACEIIs are considered stem/progenitor cells for the alveolar epithelium in homeostasis and regeneration (Hogan et al., 2014). Hence, an increased proliferation of ACEIIs via YAP/TAZ triggered by cyclic stretching could also promote the regeneration process in other organs. Accordingly, YAP/TAZ inhibition in AEIIs mice showed increased IL-1β levels and prolonged fibrotic lesions and delayed regeneration compared to normal YAP/TAZ mice (Liu et al., 2016; LaCanna et al., 2019).

In addition to AEIIS, pulmonary fibroblasts also proved to be mechano-sensitive and YAP/TAZ signaling could trigger myofibroblast differentiation and activation. Pulmonary fibroblasts responded to an increase in matrix stiffness to a pathophysiological level with nuclear localization of YAP/TAZ (Liu et al., 2015) (Figure 2E). Overexpression of YAP/TAZ in pulmonary fibroblasts resulted in increased fibrogenic potential via an enhanced proliferation rate and matrix synthesis (Liu et al., 2015). Blocking YAP/TAZ signaling in pulmonary fibroblasts reversed the deposition, contraction, and stiffening of the matrix *in vitro* and was able to reverse bleomycin-induced pulmonary fibrosis *in vivo* (Haak et al., 2019). These findings suggest that YAP/TAZ might be an important player during the anti-inflammatory response leading to ECM formation, but also, if not temporal limited, to fibrosis. Therefore, YAP/TAZ could be an attractive target to control the regeneration process by downregulating fibrotic processes and directing the healing process toward regeneration rather than toward scarring.

### Immune Cells as Mechano-Transducer in Inflammation and Regeneration

Alternatively, the immune cells might directly sense mechanical properties themselves and respond with pro-inflammatory reactions. A possible way how cells perceive forces such as shear, stretch, osmotic swelling and shrinkage, stiffening and surface expansion is via the transient receptor potential vanilloid 4 (TRPV4). When activated, TRPV4 causes a Ca^2+^ influx and initiates Ca^2+^ mediated signaling cascades (Strotmann et al., 2000; Vriens et al., 2004; Wu et al., 2007; Shin et al., 2012; Jo et al., 2015; Baratchi et al., 2016; Lee et al., 2019; Rosenbaum et al., 2020). Signaling via TRPV4 in VILI was also shown to activate macrophages, increase phagocytosis and caused secretion of pro-inflammatory mediators such as reactive oxygen species (ROS) and nitrogen species (NOS) (Hamanaka et al., 2010). Overall, this suggests that increased matrix stiffness as a result of inflammation, is sufficient to increase pro-inflammatory macrophage activity (Okamoto et al., 2018; Sridharan et al., 2019). Interestingly, there might be a cross-talk between lipopolysaccharide (LPS) mediated activation of macrophages via TLR4 and TRPV4. Blocking TRPV4 signaling prevented LPS-induced acute lung injury and resulted in lower levels of pro-inflammatory molecules such as TNF-α, IL-6, and ROS (Li et al., 2019). Moreover, TRPV4 may lead to a change in the TLR4 signaling pathway, that promotes phagocytosis and bacterial clearance (Scheraga et al., 2020). This nicely demonstrates that there is a crosstalk between pro-inflammatory pathways (TLR4) and mechanosensitive pathways (TRPV4).

PIEZO1 is another mechanosensitive Ca^2+^ ion channel involved in mechanically triggered inflammation. PIEZO1 is highly expressed on macrophages and other immune cells enabling them to sense omnidirectional pressure (Tolar and Wack, 2019). Cyclic hydrostatic pressure can activate an inflammatory response in macrophages and monocytes via PIEZO1 (Solis et al., 2019). Mechanistically, cyclic pressure activates PIEZO1 Ca^2+^ influx in monocytes, thereby increasing the expression and secretion of endothelin1 (ET1) (Solis et al., 2019). ET1 then signals back in an autocrine manner, stabilizing hypoxia-inducible factor 1-alpha (HIF-1α), which ultimately increases the expression and secretion of the chemoattractant CXCL2 in monocytes (Solis et al., 2019). Finally, neutrophils migrate from the blood along the CXCL2 gradient into the lung and initiate a pro-inflammatory response (Solis et al., 2019) (Figure 2F). In summary, this shows that mechanical stimuli play a role in the activation of innate immune cells and in the case of PIEZO1 and TRPV4 can trigger an inflammatory response.

## Heart Inflammation Is Modulated Via Immune-Mechanics

**Figure 3:**
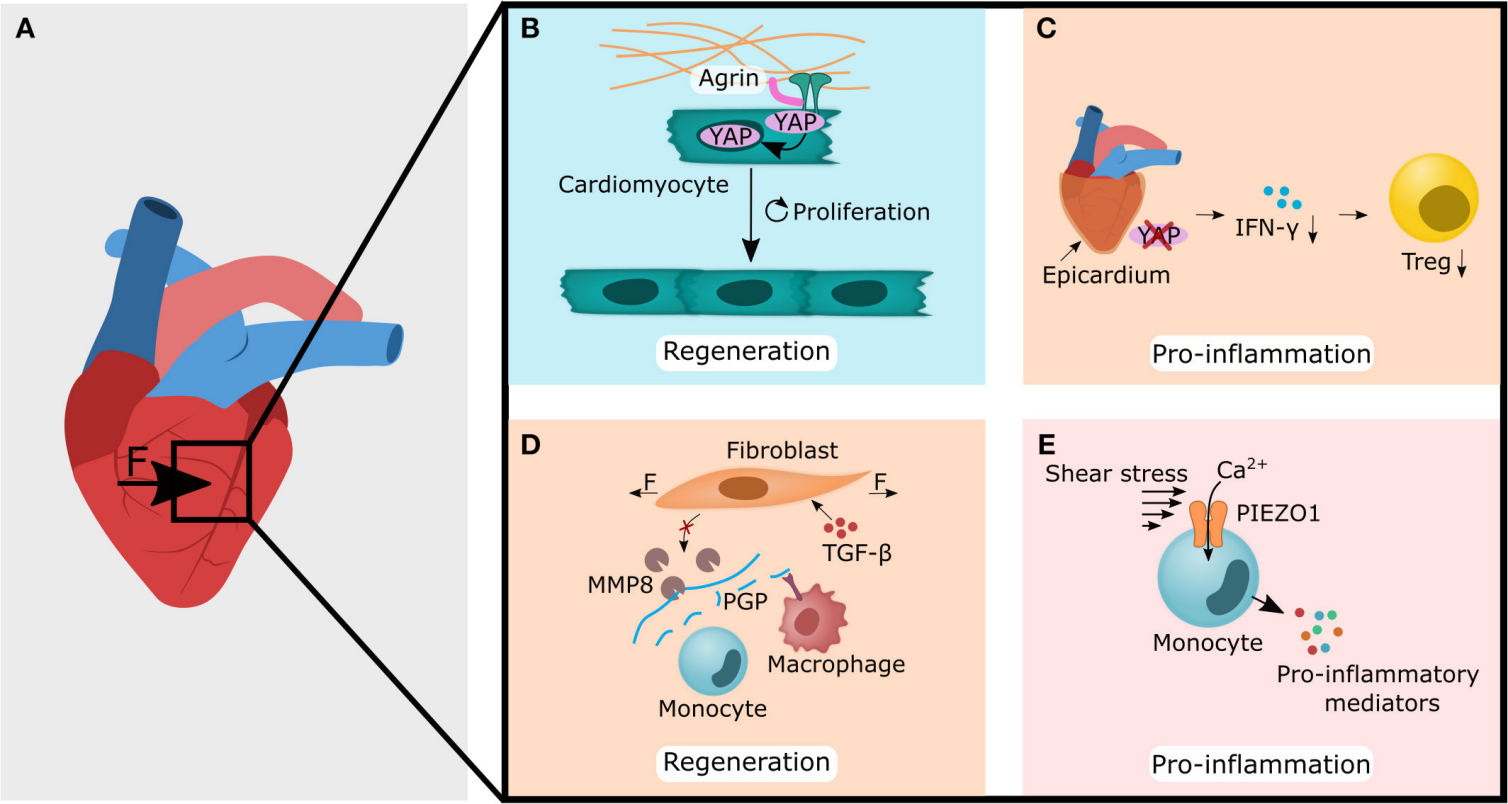
Forces acting on heart tissue changes the extracellular matrix (blue panel), and are perceived by non-hematopoietic (light orange) and immune cells (pink panel), thereby leading to regenerative or tissue destructive processes. **(A)** Forces that are caused for example by transverse aortic constriction may lead to the cellular responses shown in **(B–E)**. **(B)** The extracellular component agrin increases the stability of the ECM-integrin interaction, resulting in enhanced nuclear localization of YAP/TAZ in cardiomyocytes. Nuclear YAP/TAZ localization may then increase the proliferation rate of cardiomyocytes. **(C)** YAP/TAZ knock out in the epicardium leads to lower levels of INF-γ and a decrease of regulatory T cell (Treg) numbers. **(D)** Transverse aortic constriction leads to higher forces perceived by fibroblasts. Together with higher TGF-β levels, this decreases the MMP8 levels and the MMP8 cleavage product proline-glycin-prolin (PGP). Subsequently, the reduced PGP levels downregulate the recruitment and activation of monocytes and macrophages. **(E)** High shear stress during aortic valve stenosis leads to Ca^2+^ influx in monocytes, upon which they react with the secretion of pro-inflammatory mediators, such as IFN-β1, IL-1β, IL-1ra, IL-6, and IL-12. The YAP symbol in the illustrations is representative for YAP and TAZ. IL, interleukin; IFN, interferon; MMP8, Matrix metallopeptidases 8; TAZ, WW domain-containing transcription regulator 1; TGF-β, transforming growth factor β; YAP, yes-associated protein 1.

In contrast to most other tissues, heart tissue appears to preferentially form fibrotic tissue rather than new muscle tissue in response to damage (Russo et al., 2019). It is assumed that the fibrotic tissue spatially confines the injured area and thus prevents further damage of healthy tissue under constant mechanical stress (Russo et al., 2019). In contrast to healthy tissue, scar and fibrotic tissue have a different structure in their ECM and are probably also more readily formed (Willyard, 2018). Here, the fastest possible restoration of “functional tissue” seems to be of crucial importance, and a regeneration pause is not an option. Consequently, the preferred strategy of forming fibrotic tissue after injury allows the maintenance of structural support at the expense of an active contribution to the dynamic function of the heart. The use of mechanical unloading strategies via axillary pumps, primarily used as a mechanical circulatory support in cardiogenic patients, have been shown to boost endogenous repair in form of reduction in immune cell presence, modulation of the ECM and cardiac metabolism, which go along with a drop in integrin expression (Spillmann et al., 2019; Tschöpe et al., 2019). This example from cardiac regeneration (or lack thereof) illustrates how local immune (im-)balance regulate tissue maintenance and fibrosis after injury while being under constant mechanical stress (Figure 3A).

### ECM-Components as Mechano-Transducer in Inflammation

Some ECM components in the heart tissue can serve as mechano-transducers. For example, cells responded to a stiffer ECM with increased expression of heparan sulfate proteoglycan agrin (Chakraborty et al., 2017). Since its discovery in the early 90s, many functions of agrin have been identified. The most prominent and one of the earliest discoveries was the role of agrin as a key regulator of the formation of postsynaptic structures at the neuromuscular junction (Ruegg and Bixby, 1998). Surprisingly, agrin was also found on the surface of T cells and contributes as co-stimulatory signal, as well as to the strengthening of the immunological synapse between T cells and antigen-presenting cells (APCs) (Bezakova and Ruegg, 2003). Agrin was also found in the growth plate of bone and was shown to be highly expressed by chondrocytes (Hausser et al., 2007). It was later suggested that agrin is also an important extracellular component of the ECM-integrin-FAK axis and therefore helps to connect the cellular cytoskeleton to the ECM to ensure proper force transmission and matrix stiffness transfer (Haak et al., 2019). Moreover, agrin appears to be essential for the regenerative abilities of the neonatal mouse heart by promoting the division of cardiomyocytes via YAP- and ERK-mediated signaling (Bassat et al., 2017) (Figure 3B). *In vivo*, a single administration of agrin in adult mice after myocardial infarction promoted cardiac regeneration by pleiotropic effects such as cardiomyocyte proliferation, immune modulation and angiogenesis (Bassat et al., 2017). For example, agrin mediated signaling has been shown to cause increased actin cytoskeletal rearrangements, with the result that agrin deficiency impaired phagocytosis in macrophages and ultimately the clearance of tissue debris after injury (Mazzon et al., 2012). Adult mammalian hearts generally have a very low regenerative capacity, likely due to the inability of fully differentiated cardiomyocytes to proliferate (Kikuchi and Poss, 2012; Bigotti et al., 2020). Therefore, therapies that promote the proliferation of cardiomyocytes via YAP/TAZ-signaling such as the administration of the ECM-component agrin, are an exciting new approach, whose full potential, including its effect on immune cells, could be the focus of further research.

### Non-hematopoietic Cells as Mechano-Transducer in Inflammation and Regeneration

Current findings suggest that inflammation is likely to have both negative and positive effects on regeneration, with an initial pro-inflammatory phase being necessary to start the regeneration process, but an uncontrolled and prolonged inflammatory response is detrimental (Coggins and Rosenzweig, 2012). It is therefore not surprising that macrophages and monocytes are essential for a good healing outcome (Morimoto et al., 2006; Van Amerongen et al., 2007; Leblond et al., 2015). One way to promote the local accumulation of monocytes is through cyclic stretching. For example cyclic stretching of rat neonatal cardiomyocytes resulted in increased TLR4 expression and consequently to more TLR4 mediated adhesion of monocytes to cardiomyocytes (Shyu et al., 2010). Additionally, it has been shown that cyclic stretching of rabbit cardiomyocytes increased IL-18 expression (Yoshida et al., 2014). IL-18 is a potent growth factor that induces cardiomyocyte hypertrophy *in vitro* (Chandrasekar et al., 2005) and myocardial hypertrophy *in vivo* (Reddy et al., 2010). However, it is believed that the initiation and control of inflammation is regulated by cells in the epicardium such as fibroblast and not by myocytes (Ramjee et al., 2017). For example, *in vitro* mechanical stretching, which mimics cardiac dilatation during heart failure, induces fibroblast activation and stimulates not only the production of ECM but also the production of chemokines, such as the monocyte chemoattractant protein (MCP)-1 and MCP-3, and triggers typical inflammatory pathways (Lindner et al., 2014).

Although an improved healing outcome by YAP/TAZ signaling in cardiomyocytes is often attributed to increased myocyte proliferation (Lin et al., 2014; Leach et al., 2017; Wang et al., 2018), YAP/TAZ may also play an immunomodulatory role in regeneration. It has been found that the loss of YAP/TAZ in the epicardium led to an enhanced fibrotic response and was associated with a decrease in IFN-y expression, which is a direct transcriptional target of YAP/TAZ (Ramjee et al., 2017) (Figure 3C). Interestingly, IFN-γ has also been described to downregulate fibrotic processes. For example, IFN-γ was shown to increase the number of regulatory T cells after myocardial infarction and lead to less fibrosis (Ramjee et al., 2017) (Figure 3C). Furthermore, IFN-γ counteracts TGF-ß induced activation of myofibroblasts and leads particularly to the expression of chemokines attracting pro-inflammatory monocytes (Pappritz et al., 2018). Thus, the role of IFN-γ seems to be ambivalent and might also depend on the presence of other cytokines and cells.

In a pressure overload model by transversal aortic constriction (TAC) the concentrations of TNF-α and IL-1β were significantly elevated after 3 days and increased levels of the chemokines MCP-1, MIP-2, and TGF-β were found after 7 days (Xia et al., 2009). In contrast to sudden trauma-induced tissue damage, TAC induced injury takes time to develop, which could explain the long span needed for upregulation of cytokines and chemokines. Nevertheless, it has been shown that mechanically induced secretion and activation of TGF-β plays an important role in the activation and transformation of fibroblasts into myofibroblast (Chen and Frangogiannis, 2013; Van Linthout et al., 2014; Pappritz et al., 2018; Steffens et al., 2020). Although myofibroblasts are required for the initial healing response, excessive and prolonged activity of myofibroblasts drives scar formation and fibrosis (Gabbiani, 2003; Darby et al., 2014). In the heart, this increased and prolonged activity of myofibroblasts might be the intrinsically preferred mode of healing after injury, to re-establish mechanical stability as quickly as possible. Recently, it has been found that the increased TGF-β levels after TAC, reduced MMP-8 expression in fibroblasts, which resulted in matrix preservation rather than matrix degradation as well as decreased macrophage-driven inflammation and attenuated cardiomyocyte apoptosis (Russo et al., 2019) (Figure 3D). MMP-8 is known to generate pro-inflammatory matrix fragment proline-glycine-proline (PGP) that promote recruitment and activation of neutrophil cells and polarize macrophages into a pro-inflammatory phenotype (Lin et al., 2008).

Whether this healing mechanism of the heart is something cell- and tissue-inherent, or whether the constant mechanical stress contributes to the preferred fibrotic healing outcome, is still to a large part elusive. Further investigations of the mechanisms involved could help to better understand fibrotic processes and lead to new therapeutic approaches that make use of the mechanical manipulation of the immune system.

### Immune Cells as Mechano-Transducer in Inflammation and Regeneration

Surprisingly, either the direct influence of mechanical stimuli on immune cells in the heart has so far been neglected, or it does not play an important role in the development of cardiac pathologies and cardiac regeneration. However, a very recently published study indicates that in aortic valve stenosis causing high shear stress, PIEZO1 can be activated on **monocytes**, resulting in calcium influx and pro-inflammatory responses, such as increased expression of IFN-β1, IL-1β, IL-1ra, IL-6, and IL-12 (Baratchi et al., 2020) (Figure 3E).

Recently, YAP/TAZ signaling in immune cells has been described to reduce fibrosis in the heart (Geng et al., 2017; McGeachy, 2017). TAZ has been found to play a regulatory role in the balance of Th17–Treg by promoting acetylation and degradation of the Treg transcription factor Foxp3 (Geng et al., 2017; McGeachy, 2017). Although Tregs produce TGF-β that promotes fibrotic processes, it is thought that Tregs have a more dominant anti-fibrotic function by down-regulating the activity of Th2 cells; one of the major cellular contributors to fibrosis (Gieseck et al., 2018; Bal and Stadhouders, 2019).

This suggests that immune cells are also able to sense mechanical stimuli directly in heart tissue, similar to findings in lung tissue. To what extent this plays a role in initiating an inflammatory response in other organs could be a promising focus of further research.

## Skin Inflammation Is Modulated Via Immune-Mechanics in Wound Healing

**Figure 4:**
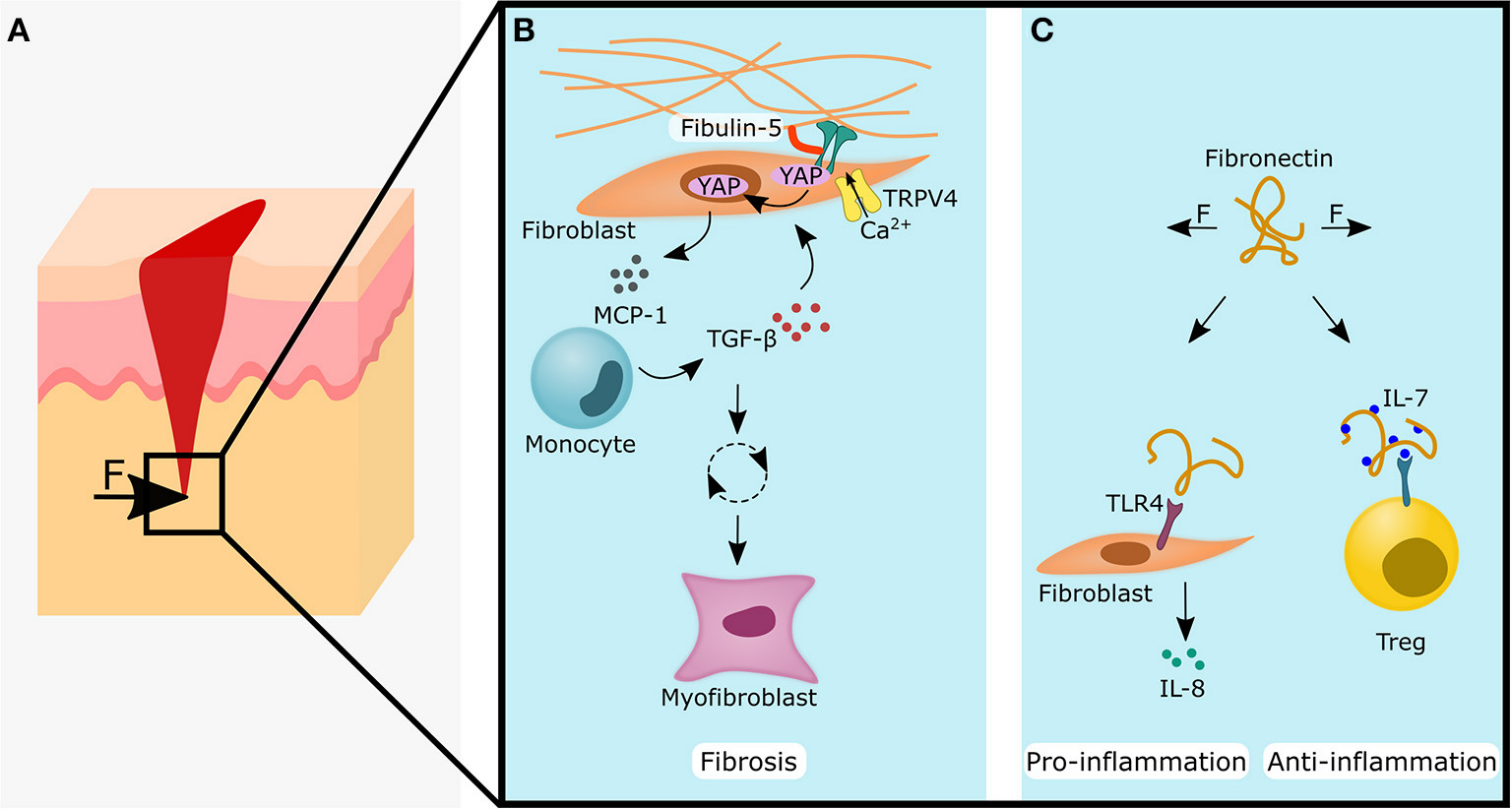
Forces acting on skin tissue during wound healing influences fibrotic, pro-inflammatory and anti-inflammatory processes by changing the extracellular matrix properties (blue panels). **(A)** Forces acting on a skin wound may lead to the cellular responses shown in **(B** and **C)**, **(B)** Fibulin-5 increases the stability of the ECM-integrin connection and the formation of focal adhesions. This might promote nuclear YAP/TAZ localization and together with the additional input of TRPV4, this leads to the secretion of monocyte chemoattractant protein (MCP)-1. Monocytes will migrate along the MCP-1 gradient and secrete TGF-β, which acts back onto the fibroblast. If unchecked, this starts a vicious cycle leading to myofibroblast differentiation and fibrosis. **(C)** Left side: Stretched fibronectin conformation can function as a Damage Associated Molecular Pattern (DAMP) by biding to TLR4 on fibroblasts and lead to the secretion of the pro-inflammatory cytokine IL-8. Right side: Conversely, the stretched fibronectin conformation shows a higher binding affinity of IL-7. Higher levels of IL-7 can increase the stability and numbers of regulatory T cells (Tregs), which is often associated with an anti-inflammatory response. The YAP symbol in the illustrations is representative for YAP and TAZ. IL, interleukin; TAZ, WW domain-containing transcription regulator 1; TGF- β, transforming growth factor β; TLR4, toll-like receptor 4; TRPV4, transient receptor potential vanilloid 4; YAP, yes-associated protein 1.

The skin is surprisingly mechano-sensitive (Kwon et al., 2018). Probably the most prominent example is the thickening of the epidermis and callus formation after repeated loading at the same site (Goldstein and Sanders, 1998; Silver et al., 2003; Carlson, 2006; Kim et al., 2010). Thickening of the epidermis is suggested to protect the underlying tissue from the excessive stress and to contribute to a better distribution of forces (Thomas et al., 1985; Kim et al., 2010). Impressively, Karl Langer recognized as early as 1861 from the observation that circular hole punches in the skin became elliptical, that skin had oriented pre-tension lines (Langer, 1978a,b,c). Additionally, in the skin, collagen bundles were found to be oriented along the main tensile load-lines/Langer lines (Wilhelmi et al., 1999; Silver et al., 2003). As a result, surgical incisions are often performed parallel to the Langer lines instead of perpendicular to reduce scarring (Wilhelmi et al., 1999). Besides the passive pre-tension of the skin, active stress generation and contraction, mainly produced by (myo-)fibroblasts, are important for wound closure and reduced scarring. It has been proposed to link mechano-transduction to various skin pathologies such as keloids and hypertrophic scars, and subsequently targeting mechanoreceptors has already been suggested as novel treatment options (Ogawa and Hsu, 2013). For example, pressure has been found to positively influence the wound healing phase by upregulating the apoptosis of myofibroblasts (Costa et al., 1999; Renò et al., 2003). At the beginning of the wound-healing phase, myofibroblasts are thought to have a positive effect by increasing ECM production and its contractile function bringing the two wound edges back together again (Hinz et al., 2012; Humphrey et al., 2014). However, it has been found that excessive matrix production and stiffening of the wound could initiate a vicious cycle leading to prolonged and excessive myofibroblast activity, which ends in fibrosis and hypertrophic scaring (Hinz, 2007; Darby et al., 2014). Interestingly, the mechanical tension applied to a healing wound was already sufficient to produce hypertrophic scarring in mice (Aarabi et al., 2007). Cyclic tensioning of keratinocytes was shown to increase proliferation, while cyclic compression downregulated cell division of keratinocytes (Silver et al., 2003). Thus, it has been known for a relatively long time that the mechanical environment and properties are playing a crucial role for skin wound healing (Tomasek et al., 2002; Silver et al., 2003) (Figure 4A). However, the relationship between the mechanical environment and the immune system, which regulates the healing response, has only recently become the focus of research.

### ECM-Components as Mechano-Transducer in Inflammation

The compression of hypertrophic scars was found to release significantly higher levels of IL-1β compared to normal scars (Renò et al., 2003). This could indicate that the cells within the hypertrophic scar are already pre-sensitized to mechanical stimuli or that more immune cells could be found in the hypertrophic scar. It has been found that the ECM component fibulin-5 plays an important role in inflammation-associated skin pathologies (Nakasaki et al., 2015). The loss of fibulin-5 not only led to a decrease in tissue stiffness, but also reduced the inflammatory response (Nakasaki et al., 2015) (Figure 4B). The increase in stiffness from 2 to 5 kPa was sufficient to enhance the pro-inflammatory response of dermal fibroblasts with increased expression of the chemoattractants CXCL5, CXCL10, and CXCL16, and resulted in increased numbers of macrophages, T cells and mast cells (Nakasaki et al., 2015). Targeting fibulin-5 could prevent the stiffening of skin ECM and could be an attractive target to stop the fibrotic vicious cycle. Another important ECM component that converts mechanical stimuli into an inflammatory response is fibronectin. Dermal fibroblasts have been shown to recognize stretched fibronectin as damage associated molecular patterns (DAMPs), which leads via the TLR4/NF-κB pathway to increased IL-8 expression (Zheng et al., 2020) (Figure 4C). Moreover, stretched fibronectin showed a higher binding affinity for IL-,7 and local accumulation of IL-7 may play an important role in the regulation of the immune response (Ortiz Franyuti et al., 2018) (Figure 4C). IL-7 was found to be essential for the maintenance of memory Tregs (mTregs) in the skin (Gratz et al., 2013).

### Non-hematopoietic Cells as Mechano-Transducer in Inflammation and Regeneration

It is suggested that the focal adhesion kinase (FAK) connects mechanical forces with skin fibrosis via inflammatory signals. In fibroblasts, FAK became activated after a skin injury and its activity can be potentiated by mechanical stretching (Wong et al., 2012). An elevated FAK signaling in fibroblasts then amplified MCP-1/CCL2 signaling and subsequently led to the migration of monocytes toward the wound and the secretion of inflammatory cytokines such as TGF-β; which possibly triggers a vicious cycle that ends in a hypertrophic scar (Wong et al., 2012). FAK signaling in dermal fibroblasts also proved to be an important player in pathological angiogenesis in hypertrophic scarring (Gao et al., 2019). Recently, TRPV4 was discovered as an important mediator in skin pathologies. In concert with TGF-β, TRPV4 was found to be essential for matrix-stiffness mediated differentiation of dermal fibroblasts into myofibroblasts (Sharma et al., 2017) (Figure 4B) and epithelial-mesenchymal transition (EMT) of epidermal keratinocytes (Sharma et al., 2019). Higher levels of TGF-β could also be triggered by increased ECM stiffness: In human dermal fibroblasts, YAP/TAZ works in conjunction with the transcription factor activator protein (AP)-1 to regulate TGF-β signaling (Qin et al., 2018). Accordingly, YAP/TAZ has already been proposed as an attractive new therapeutic target for reducing fibrosis (Duscher et al., 2014).

In summary, higher ECM stiffness, possibly due to higher concentrations of fibulin-5, increases TGF-β levels and monocyte counts, which promotes the differentiation of dermal fibroblasts into myofibroblasts and EMT of keratinocytes, and ultimately leads to skin fibrosis or hypertrophic scarring. This shows that the increased ECM-stiffness in fibrosis might not only be a consequence of the inflammatory process, but rather plays an active role in initiating the inflammatory process.

## Discussion

### The Described Immune-Mechanical Principles in the Lung, Heart, and Skin May Also Play a Role in Bone Regeneration

**Figure 5:**
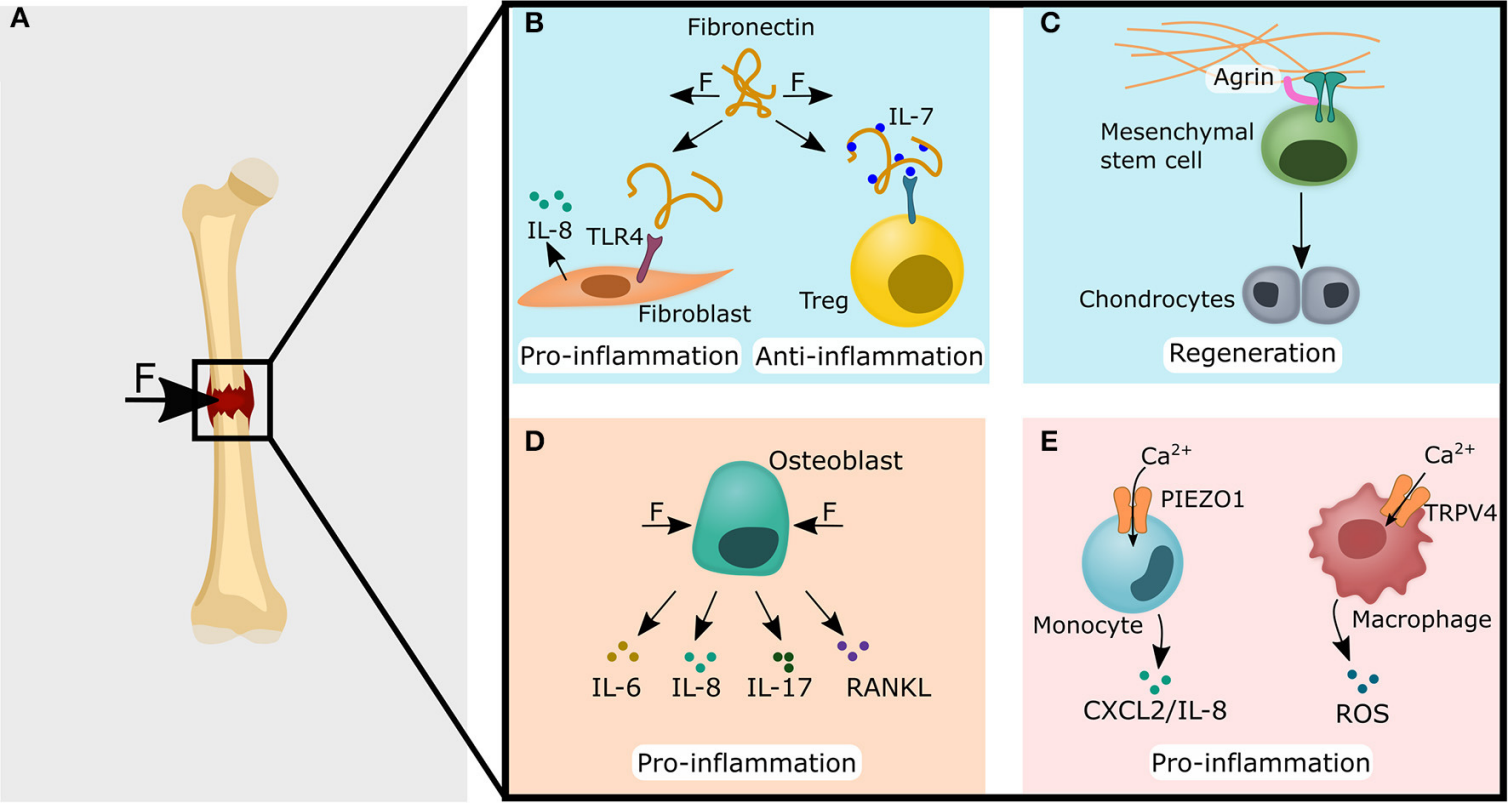
Common immune-mechanical principles found in the lung, skin and heart could potentially impact the fracture healing cascade via ECM modulation (blue panels), non-hematopoietic cells (light orange panel) and immune cells (pink panel). **(A)** Forces acting on and within the fracture gap might lead to the cellular processes described in **(B–E)**. **(B)** Fibronectin can be found in the growth plate and during new bone formation. Fibronectin could have similar roles in the bone as it was found for lung tissue, where stretched fibronectin conformation promoted a pro-inflammatory reaction by acting as a DAMP and increased the release of IL-8. Conversely, a stretched fibronectin conformation might also locally increase IL-7 levels, which leads to a higher regulatory T cell stability and numbers. **(C)** Agrin has been shown to promote heart regeneration and could also promote bone regeneration via osteochondral healing since Agrin mediated signaling leads to increased chondrogenic differentiation of mesenchymal stem cells. **(D)** The compression of osteoblasts has already been found to increase pro-inflammatory mediators such as IL-6, IL-8, IL-17, and Receptor Activator of NF-κB Ligand (RANKL). **(E)** Similar to the findings in the lung, Ca^2+^ influx via PIEZO1 in monocytes could lead to the secretion of CXCL2 in bone and attract polymorphonuclear leukocytes (left site). In macrophages, the calcium-mediated signaling via TRPV4 has been found in lung tissue to lead to reactive oxygen species (ROS) secretion. A comparable mechanism could potentially also lead to the ROS levels found in the fracture hematoma (right site). CXCL, C-X-C motif ligand; DAMP, damage-associated molecular pattern; IL, interleukin; Treg, regulatory T cell; TLR4, toll-like receptor 4; TRPV4, transient receptor potential vanilloid 4.

Forces acting on the fracture hematoma/callus (Figure 5A) likely influences the healing outcome by regulating the immune response via: ECM components (Figures 5B,C), indirectly via the sensation of forces by osteoblasts and subsequent cytokine release (Figure 5D), or forces are directly sensed by immune cells (Figure 5D). Fibronectin is an important and ubiquitous component of the ECM with structural and functional roles in homeostasis and regeneration (To and Midwood, 2011). So far, fibronectin has also been found in the growth plate of bone and during new bone formation, which may promote osteoblast differentiation and the mineralization process (Yang et al., 2020). Although higher levels of fibronectin were found in osteoporotic patients, the downregulation of a fibronectin receptor (β_1_-integerin) on the cell surface reduced fibronectin mediated signaling in osteoblast and might explain the loss of bone despite higher fibronectin concentrations (Yang et al., 2020). Whether these higher fibronectin levels contribute to the osteoporotic pathology via immunomodulatory mechanisms, has not yet been fully elucidated. However, lung cells and dermal fibroblasts have been shown to bind the stretched fibronectin conformation via TLR4, which led to increased IL-8 levels (Cho et al., 2020; Zheng et al., 2020). Since IL-8 also seems to play an important role in bone healing (Lin et al., 2019), mechanical manipulation of the fibronectin conformation may also be an attractive therapeutic target (Figure 5B). Furthermore, as in the skin, the mechanical niche during fracture healing could influence the healing outcome via the concentration of IL-7 by the conformational regulation of fibronectin. Subsequent, as described in the skin, this could promote the anti-inflammatory phase via the stabilization of memory Tregs (Gratz et al., 2013) (Figure 5B).

Another ECM component that is found in bone and is involved in the regulation of the immune system is agrin (Bezakova and Ruegg, 2003; Hausser et al., 2007). In the heart, the administration of agrin was able to increase the proliferation rate of cardiomyocytes via YAP/TAZ signaling, resulting in improved healing response (Bassat et al., 2017). Potentially, this therapeutic approach could also be translated to enhance bone healing. It has already been found that agrin plays an important role in osteochondral regeneration (Eldridge et al., 2020) (Figure 5C). Since critical-sized bone defects heal via endochondral ossification (Dennis et al., 2015), which involves the formation of cartilage and its subsequent transformation into bone, promoting cartilage formation to heal critical-sized bone defects could be an appropriate approach and is already a focus of current research (Petersen et al., 2018; Lin et al., 2019).

Moreover, the mechanosensitive ion channels PIEZO1 and TRPV4 might also play a role in bone healing. Similar to the findings in lung tissue, IL-8 also attracts PMNs in bone tissue, which have been shown to be a critical player in bone fracture healing (Kovtun et al., 2016; Lin et al., 2019). It could be hypothesized that not only the lung monocytes perceive cyclic pressure via PIEZO1, but also the monocytes in bone healing, which would lead to a contribution to the IL-8 levels found in the fracture hematoma (Kolar et al., 2011) (Figure 5E).

In addition, signaling via TRPV4 in lung macrophages has been found to increase ROS levels (Hamanaka et al., 2010; Li et al., 2019) (Figure 5E). It could be hypothesized that similar processes may also occur during bone healing. ROS has been shown to interfere with the osteogenic potential of MSCs and the administration of antioxidant resulted in improved bone regeneration *in vivo* (Geißler et al., 2013). However, the situation might be more complex. Recently, it was found that MSCs embedded in a 3D collagen hydrogel might sense nanovibrations via integrins and mechanosensitive ion channels, that increased their osteogenic differentiation (Tsimbouri et al., 2017). Later, the same authors suggested that MSCs stimulated with nanovibrations produce ROS during osteogenic differentiation, but the ROS themselves do not appear to be a driver of osteogenesis (Orapiriyakul et al., 2020). Whether ROS is only a byproduct during bone healing or whether it takes an active role in this process is still elusive and further studies are required. If ROS does actively influence bone healing, it would be interesting to investigate, whether macrophages in the bone fracture hematoma also sense mechanical stimuli via mechanosensitive ion channels, such as TRPV4, and also produce ROS as they do in lung injuries.

During bone healing, the local mechanical niche changes a lot. The fracture hematoma is soft and relatively dense. While in the subsequent healing phases the stiffness increases steadily. This might have different implications on the cellular behavior of non-hematopoietic and immune cells. On soft and confined substrates, cells tend to show spherical morphology and cytoplasmic localization of YAP/TAZ, while more open and stiff substrates allows for cytoskeletal re-arrangements, cell spreading and nuclear localization of YAP/TAZ (Panciera et al., 2017). Interestingly, mechanical confinement was not only found to restrict cell spreading, but also reduced the osteogenic differentiation potential of MSCs via a lack of TRPV4 activation (Lee et al., 2019). Also osteoclasts activity is dependent on cytoskeletal re-arrangements (Takayanagi, 2007). Recent evidence also points out, that T cells are dependent on cytoskeletal re-organization for an efficient immunological synapse formation and T-cell activation (Colin-York et al., 2019; Blumenthal and Burkhardt, 2020). Whether this has also implications in a non-antigen specific, sterile inflammation process, as found during regeneration, could be the scope of future research.

These examples encourage that also during bone regeneration the immune response is modulated by the ECM composition and the mechanical niche. In particular, the ECM components fibronectin and agrin could also have immunomodulatory functions in bone, and TRPV4 and PIEZO1 on macrophages could be important mechano-transducers and thereby influence the outcome of bone healing.

### Summary

**Figure 6:**
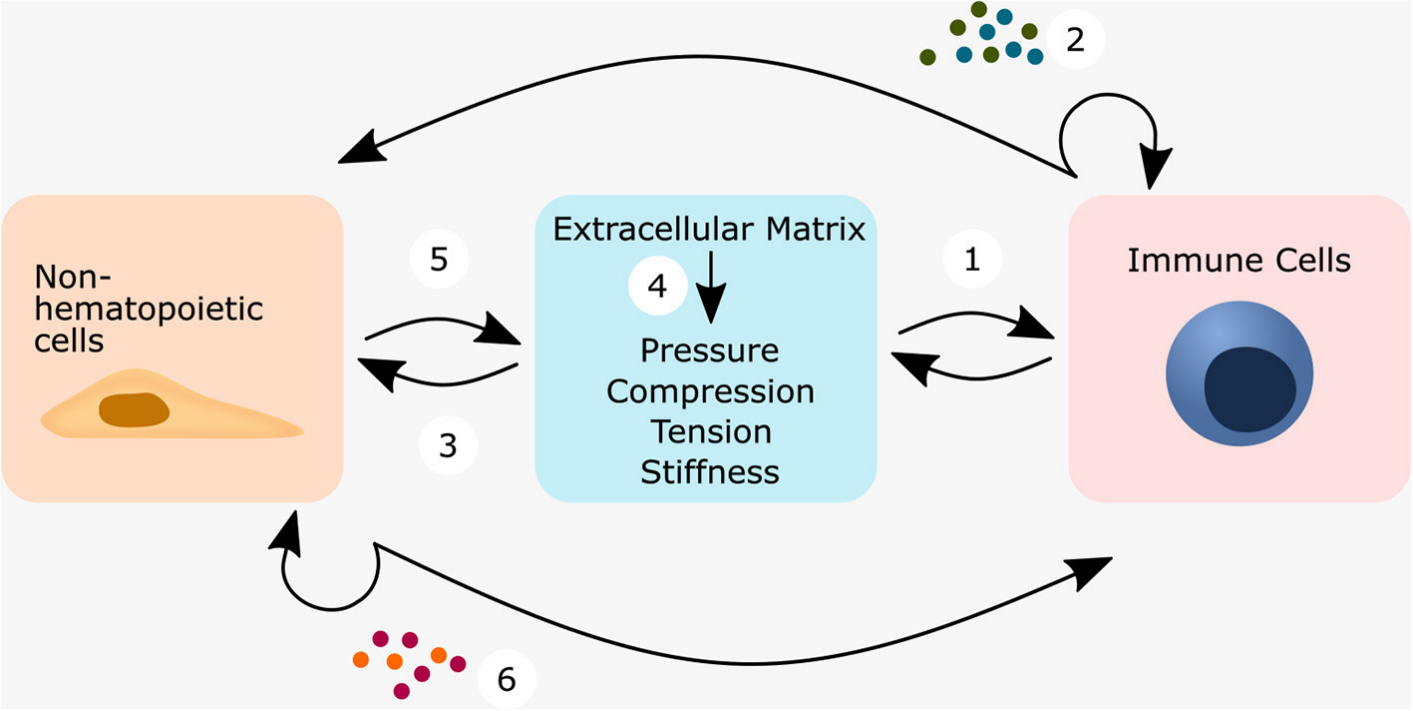
Mechanically or ECM triggered crosstalk between non-hematopoietic cells—immune cells—ECM regulates cell behavior. Immune cells can respond either directly or indirectly to mechanical forces. When immune cells sense the mechanical niche directly (1), they can transmit this information by the cytokine secretion, which either acts in an autocrine manner or on non-hematopoietic cells (2). This connects immune cells within a wound environment not only with each other, but also to non-hematopoietic cells such as smooth muscle cells, fibroblasts, epithelial cells and endothelial cells. Non-hematopoietic cells might then start to secrete ECM-components and change the ECM-composition (3) and subsequently its biophysical properties (4). This could close a feedback loop, and immune cells sense the altered ECM-composition/biophysical cues. Depending on the secreted cytokines of the immune cells, this feedback could be the first step into a vicious cycle or alternatively the first step toward a successful restoration of homeostasis. In the indirect case, tissue-resident cells embedded in the ECM first perceive the mechanical stimuli (5), translate them into a cellular response and might then transmit the stimuli to nearby immune cells by secreting cytokines and/or chemokines (6).

The understanding of regeneration has come a long way. Today, both the adaptive and the innate immune system are now considered to play a central role in healing and regeneration (Medzhitov, 2008; Julier et al., 2017). Although Julius Wolff and Karl Langer have already in the 19th century put forward the hypothesis that mechanical forces contribute significantly to the biological functions of tissue adaptation and healing (Langer, 1978a,b,c; Wolff, 1986), it is only recently that the mechanical environment and its influence on a central part of the biological component, the local immune response, has been investigated. Immunomodulatory mechano-transduction can either occur directly in immune cells or indirectly in non-hematopoietic cells. In either case, there is extensive crosstalk between immune and non-hematopoietic cells to determine the appropriate response for a given situation (Figure 6). The crosstalk and communication cycle could be proposed as follows: Immune cells perceive the mechanical niche at least by means of integrin, PIEZO1 and TRPV4 [Figure 6(1)] and then release cytokines and chemoattractants, which act on nearby immune cells or on non-hematopoietic cells [Figure 6(2)]. In response, non-hematopoietic cells alter the components and structure of the ECM [Figure 6(3)], which is often accompanied by a change in the mechanical niche [Figure 6(4)]. Alternatively, non-hematopoietic cells are assumed to perceive the mechanical niche at least by means of integrins, PIEZO1 and TRPV4 [Figure 6(5)] and then transmit the information of the mechanical niche to immune cells or non-hematopoietic cells via cytokines and chemoattractants [Figure 6(6)]. This might then alter the activation, differentiation, proliferation and migration of the cells.

In most situations, unphysiological mechanical stimuli or mechanical overstimulation resulted in the release of common pro-inflammatory mediators. Specific ECM components are actively involved in immune regulation, either by acting as DAMPs [LMW-HA (Jiang et al., 2005; Black et al., 2013), fibronectin (Cho et al., 2020), and biglycan (Schaefer et al., 2005)], or by stabilizing ECM-cell interactions [fibulin-5 (Nakasaki et al., 2015), and agrin (Haak et al., 2019)].

In all the tissues described, non-hematopoietic cells often respond to abnormal mechanical stimuli by secreting chemoattractants and cytokines that lure immune cells out from the vasculature and in most cases the secreted cytokines create a pro-inflammatory niche. Within the cell, calcium mediated signaling pathways, FAK and YAP/TAZ are often involved in the translation of the perceived mechanical environment into a cellular response. It is already known that YAP/TAZ plays an important role in the regulation of cell proliferation, and a targeted manipulation of this signaling pathway could help to increase the number of cells after injury (Moya and Halder, 2019). In addition, YAP/TAZ signaling in cardiomyocytes and AEIIs has been found to reduce fibrosis (Liu et al., 2016; Ramjee et al., 2017; LaCanna et al., 2019). In contrast, YAP/TAZ signaling in fibroblasts has been shown to promote fibrosis in the lung, and skin (Liu et al., 2015; Qin et al., 2018; Haak et al., 2019). This indicates that YAP/TAZ signaling can promote regeneration as well as cause fibrosis and a cell specific therapy approach may be necessary to exploit YAP/TAZ signaling.

Immune cells most often perceived the mechanical environment via mechanosensitive ion channels such as PIEZO1 (Solis et al., 2019; Baratchi et al., 2020) and TRPV4 (Hamanaka et al., 2010; Li et al., 2019). The signaling via these receptors provoked at least in monocytes and macrophages the secretion of pro-inflammatory mediators. Interestingly, in most cases when immune cells sensed the mechanical stimuli themselves, they reacted with a pro-inflammatory response. This could provide a possibility for intervention where the restoration of a mechanical niche to pre-injury level could be used to downregulate a pro-inflammatory response.

### Conclusion and Future Directions

The above findings suggest that the success of using biomaterials for cell delivery or as a cell modulating *in vivo* micro-niche also depends on the mechanical environment. *In vivo*, the biomaterial is not mechanically isolated, but connected to the mechanical environment. It is conceivable that when cells are encapsulated in the biomaterial, not only the intrinsic mechanical properties of the biomaterial determine the cell behavior, but also how much of the external mechanical stimuli are transmitted through the biomaterial. Biomaterials may not only reduce the diffusion of unwanted DAMPs or pro-inflammatory mediators but may also protect cells from pathological mechanical over-stimulation. These protected cells could then help to downregulate a too pronounced pro-inflammatory reaction.

The examples described here, are possibly just the tip of the iceberg. It seems likely that the described concepts are not only found in the lung, heart, and skin, but also in various tissues throughout the body. Since immune cells are the first cells that interact with a biomaterial and often determine the amount of a foreign body response (Anderson et al., 2008), targeting specifically immune cells via mechanical cues of the biomaterial, seems to be especially promising. If we know how to take advantage of this, it could lead to promising new therapeutic approaches. However, if the local mechanical environment is not well-controlled, it could unintentionally induce pro-inflammatory and pro-fibrotic cascades, which would jeopardize the engineered regenerative effect of the biomaterial. A better understanding of mechano-sensitive principles helps to avoid these negative effects and can even be used under certain conditions to positively influence and enhance regeneration. It is tempting to speculate that the described immune-mechanical principles are an intrinsic and universal function of the immune system that has been overlooked for a long time. Therefore, in the development of the next generation of biomaterials, it might be worthwhile to move from an approach that focuses mainly on biological factors to a more comprehensive approach that also addresses the mechanical niche and its influence on the immune system.

## Author Contributions

RK compiled the literature, prepared, and wrote the manuscript. CB, SV, CT, KS-B, and GD outlined the manuscript and edited the manuscript. All authors contributed to the article and approved the submitted version.

## Conflict of Interest

The authors declare that the research was conducted in the absence of any commercial or financial relationships that could be construed as a potential conflict of interest.
